# Analysis of differential metabolites in serum metabolomics of patients with aortic dissection

**DOI:** 10.1186/s12872-024-03798-y

**Published:** 2024-04-25

**Authors:** Yun Gong, Tangzhiming Li, Qiyun Liu, Xiaoyu Wang, Zixian Deng, Lixin Cheng, Biao Yu, Huadong Liu

**Affiliations:** 1 Department of Cardiology, Shenzhen Cardiovascular Minimally Invasive Medical Engineering Technology Research and Development Center, Shenzhen People’s Hospital (The Second Clinical Medical College, Jinan University; The First Affiliated Hospital, Southern University of Science and Technology), Shenzhen, Guangdong 518020 China; 2 Luohu People’s Hospital (Shenzhen Luohu Hospital Group, The Third Affiliated Hospital of Shenzhen University), Shenzhen, Guangdong 518020 China

**Keywords:** Aortic dissection, Differential metabolites, Metabolic biomarkers, Metabolic pathways

## Abstract

**Background:**

Pathogenesis and diagnostic biomarkers of aortic dissection (AD) can be categorized through the analysis of differential metabolites in serum. Analysis of differential metabolites in serum provides new methods for exploring the early diagnosis and treatment of aortic dissection.

**Objectives:**

This study examined affected metabolic pathways to assess the diagnostic value of metabolomics biomarkers in clients with AD.

**Method:**

The serum from 30 patients with AD and 30 healthy people was collected. The most diagnostic metabolite markers were determined using metabolomic analysis and related metabolic pathways were explored.

**Results:**

In total, 71 differential metabolites were identified. The altered metabolic pathways included reduced phospholipid catabolism and four different metabolites considered of most diagnostic value including N2-gamma-glutamylglutamine, PC(phocholines) (20:4(5Z,8Z,11Z,14Z)/15:0), propionyl carnitine, and taurine. These four predictive metabolic biomarkers accurately classified AD patient and healthy control (HC) samples with an area under the curve (AUC) of 0.9875. Based on the value of the four different metabolites, a formula was created to calculate the risk of aortic dissection. Risk score = (N2-gamma-glutamylglutamine × -0.684) + (PC (20:4(5Z,8Z,11Z,14Z)/15:0) × 0.427) + (propionyl carnitine × 0.523) + (taurine × -1.242). An additional metabolic pathways model related to aortic dissection was explored.

**Conclusion:**

Metabolomics can assist in investigating the metabolic disorders associated with AD and facilitate a more in-depth search for potential metabolic biomarkers.

## Introduction

Aortic dissection (AD) is a life-threatening condition caused by a tear in the inner layer of the aorta, which results in a separation of the layers of the aortic wall and subsequent formation of a true lumen and a false lumen [1, 2]. Current methods of diagnosing AD include the D-dimer test and contrast-enhanced computed tomography (CT) of the aorta. D-Dimers have high sensitivity, but extremely poor specificity, and enhanced CT requires expensive equipment and the rays cause potential harm to the patients [3]. The pursuit of early diagnosis and the identification of more effective therapeutic targets are crucial to ensuring the timely treatment of AD and guiding its therapy.

Metabolomics is an effective and widely used technology that has been increasingly employed to comprehensively profile disease progression [4]. Serum is frequently considered as a pool of metabolites and the analysis of serum metabolomics has been an efficient tool to identify potential metabolic biomarkers of diseases, that may improve early diagnosis, prognostic prediction, and personalized therapy [5]. Although metabolomics has made great progress in the diagnosis and treatment of diseases, most metabolomics studies mainly focus on acute myocardial infarction and coronary heart disease. There are few studies on metabolites of AD. Diagnosis of AD at an early stage is complicated and challenging. Potential metabolic biomarkers in AD patients are not clear.

This study was designed to explore the metabolites and metabolic pathways of patients with aortic dissection and search for potential biomarkers.

## Methods

### Patients and study design

Patients were enrolled from Shenzhen People’s Hospital between October 2019 and April 2021. Inclusion criteria were a diagnosis of acute aortic dissection, of less than two weeks duration, confirmed by aortic vascular enhanced CT. Patients were excluded with intramural hematoma of the aorta, aortic aneurysm, embolism, malignant tumor, severe infectious disease, trauma, a recent surgical procedure, and severe heart failure with left ventricular ejection fraction less than 20%. Informed consent was obtained from all patients. This study was performed under the guidance of the Helsinki Declaration and was approved by all centers.

Serum samples were collected before the aortic surgery and were immediately frozen at -80 °C for metabolomic analysis.

### Chemical and sample preparation

All chemicals and solvents were of analytical or HPLC (High Performance Liquid Chromatography) grade. Water, methanol, acetonitrile, and formic acid were purchased from CNW technologies GmbH (Dusseldorf Germany). The serum was separated after − 4 °C refrigeration for 60 min, and stored at -80 °C for metabolite analysis. Samples stored at -80 C were thawed at room temperature and 200 µL of the sample was added to a 1.5 mL Eppendorf tube with 10 µL of 2-chloro-l-phenylalanine (0.3 mg/mL) dissolved in methanol as internal standard. Then, the tube was vortexed for 10 s, and a 15 µL ice-cold mixture of methanol and acetonitrile was added to the sample. The mixtures were vortexed for 1 min, ultrasonicated at an ambient temperature of 25–28 °C for 10 min, and stored at -20 °C for 30 min. The extract was centrifuged at 13,000 rpm at 4 °C for 15 min, 1.0mL of supernatant in a brown and glass vial was dried in a freeze concentration centrifugal dryer and 15 µL mixture of methanol and water were added to each sample, vortexed for 30 s, and then kept at 4 °C for 2 min. Samples were centrifuged at 1300 rpm, 4 °C for 5 min. Then, 100 µL supernatant aliquots from each tube were collected using crystal syringes, filtered through 0.22 pm microflora and transferential vials. These were stored at -80 °C for liquid chromatography-mass spectrometry (LC-MS) analysis.

An ACQUITY UHPLC system (Waters Corporation, Milford, USA) was used to analyze the metabolic profiling in both ESI positive and ESI negative ion modes. An ACQUITY UPLC BEH C18 column (1.7 μm, 2.1 × 100 mm) were employed in both positive and negative modes. The binary gradient elution system consisted of (A) water (containing 0.1% formic acid, v/v) and (B) acetonitrile (containing 0.1% formic acid, v/v) .We separated sample by following gradient: 0 min, 5% B; 2 min, 20% B; 4 min, 60% B; 11 min, 100% B; 13 min, 100% B; 13.5 min, 5% B and 14.5 min, 5%B .The flow rate was 0.4 mL/min and column temperature was 45 ℃. All the samples were kept at 4℃ during the analysis. The injection volume was 5 µL.

Parameters of mass spectrometry were as follows: Ion source temperature, 550 °C (+) and 550 °C (−); ion spray voltage, 5500 V (+) and 4500 V (−); curtain gas of 35 PSI; declustering potential, 100 V (+) and − 100 V (−); collision energy, 10 eV (+) and − 10 eV (−); and interface heater temperature, 550 °C(+) and 600 °C (−). For IDA analysis, range of m/z was set as 25–1000, the collision energy was 30 eV.

Quality control (QC) samples were prepared by pooling aliquots of all samples and injected every 10 samples throughout the analytical run to provide a set of data from which repeatability could be assessed.

### Data preprocessing and statistical analysis

We analyze the LC-MS raw data by the orogenesis QI software (Waters Corporation, Milord, USA), with the parameters was set in 5 ppm (parts per million) ,10 ppm and 0.02 min for the precursor tolerance (PT), fragment tolerance (FT) and retention time (RT). According to three-dimensional data sets including m/Z, peaks RT and peak intensities. We acquired the Excel file which obtained filtered data. The internal standard was used for data QC reproducibility.

Metabolites were identified by progenesis QI Data Processing Software, based on public databases and self-built databases. We acquired combined data from combined positive and negative data, and imported it into the R ropls package. We conducted Principle component analysis (PCA) and orthogonal partial least-squares-discriminant analysis (O) PLS-DA to compare different of metabolic alterations among experimental groups. Overall contribution of each variable was ranked in the OPLS-DA model. Those variables with a VIP > 1 are considered relevant for group discrimination.

In this study, the default seven-round cross-validation was applied with one-seventh of the samples excluded from the material model inch round, to guard against overfitting.

The deferential metabolite was selected based on the combination of the statistically significant threshold of variable influence on projection (VIP) values obtained from the OPLS- DÁ model and p-values from a two-tailed Student’s test on the normalized peak areas, where metabolites with VIP values larger than 1.0 and *p* < 0.05 were considered a deferential metabolite.

## Results

**Fig. 1:**
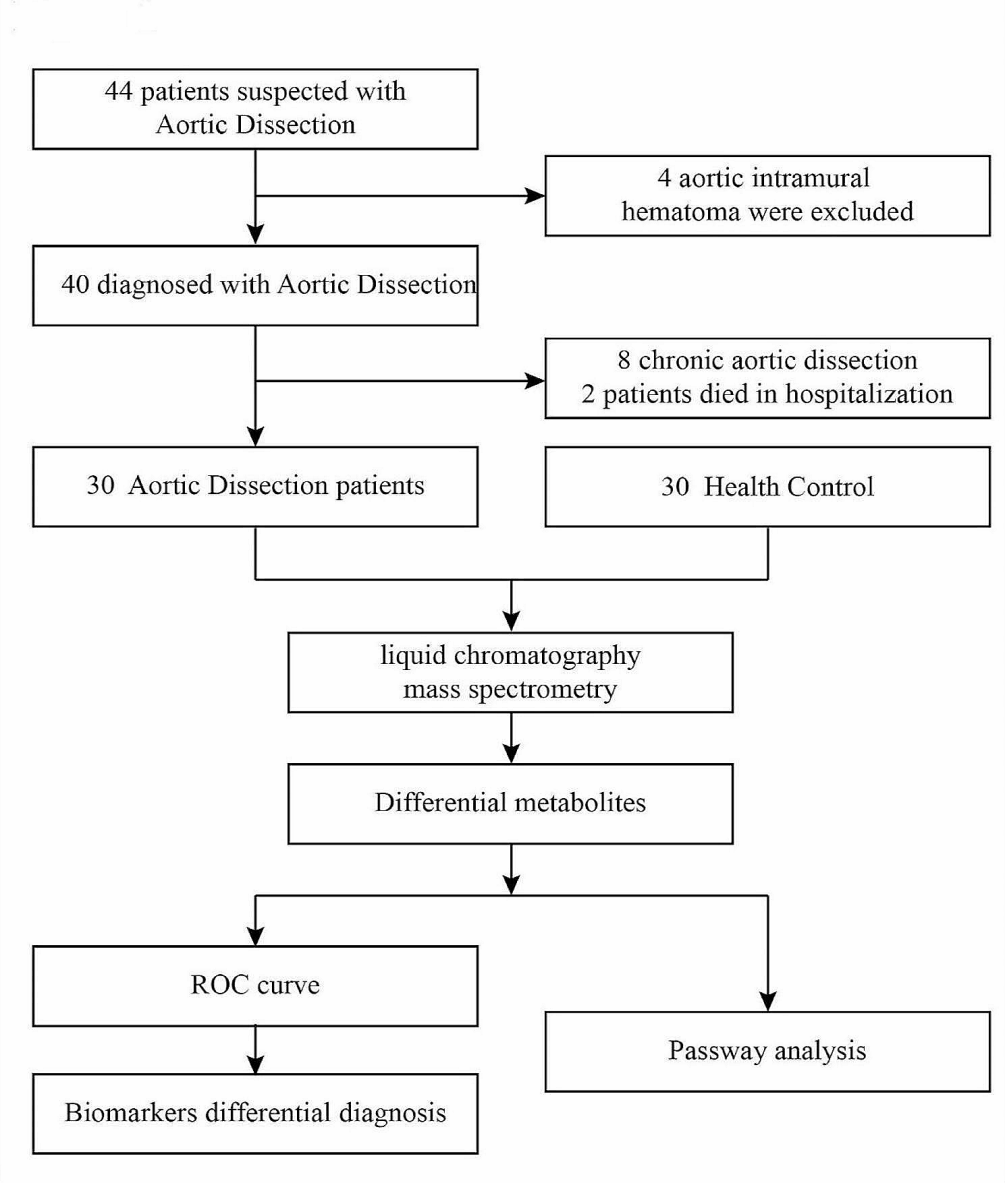
Study design: 30 AD patients and 30 healthy controls

This study followed-up 44 patients who were suspected of having AD and 30 patients diagnosed with acute AD were enrolled (see Fig. 1). Sixty samples of human serum, 30 from AD patients and 30 from healthy controls (HC), were collected for LC-MS analysis. Nontargeted profiling was performed to obtain the serum metabolic characteristics as comprehensively as possible. In this study, by employing nontargeted LC-MS platforms, the metabolic profiles of ADs and HCs could be analyzed in detail and the related disordered metabolism pathway underlying AD development investigated. A novel biomarker panel was subsequently identified and validated for differentiating ADs and HCs and its clinical practicability assessed (Fig. 1).

Baseline clinical characteristics of the AD and HC groups were compared in Table 1. Compared with HC subjects, AD patients had higher levels of heart rate and D-Dimers. There were no significant differences in age, blood pressure levels on admission, body mass index (BMI), whether hypertension and diabetes were present, cTnI, NT-pro BNP, total cholesterol, low- and high-density lipoprotein cholesterol and triglyceride levels.

**Table 1:** Baseline of patients with aortic dissection and healthy controls

	Aortic dissection (*n* = 30)	Heath Control (*n* = 30)	p Value
Male (n[%])	27 (90.0)	25 (83.3)	>0.05
Age (years)	52.93 ± 12.11	53.87 ± 12.22	>0.05
HR (bmp)	74.47 ± 19.18	83.43 ± 13.43	<0.05
SBP (mmHg)	147.40 ± 35.61	136.10 ± 32.44	>0.05
DBP (mmHg)	83.77 ± 20.65	84.30 ± 19.23	>0.05
BMI (kg/m2)	25.77 ± 4.04	24.12 ± 3.06	>0.05
Hypertension (n[%])	26 (86.7)	23 (76.7)	>0.05
Diabetes (n[%])	3 (10.0)	4 (13.3)	>0.05
Triglycerides(mmol/L)	1.60 ± 0.75	1.42 ± 0.95	>0.05
TC(mmol/L)	4.22 ± 1.23	4.01 ± 1.15	>0.05
LDL-C(mmol/L)	2.46 ± 1.09	2.36 ± 0.96	>0.05
HDL-C(mmol/L)	1.06 ± 0.33	1.08 ± 0.31	>0.05
cTnI(ng/dL)	0.07 ± 1.09	1.87 ± 0.62	>0.05
NTpro-BNP(pg/ml)	4017.34 ± 14098.03	2557.83 ± 5094.34	>0.05
D-Dimer(ug/L)	8970.75 ± 18508.39	594.64 ± 720.81	<0.05

**Fig. 2:**
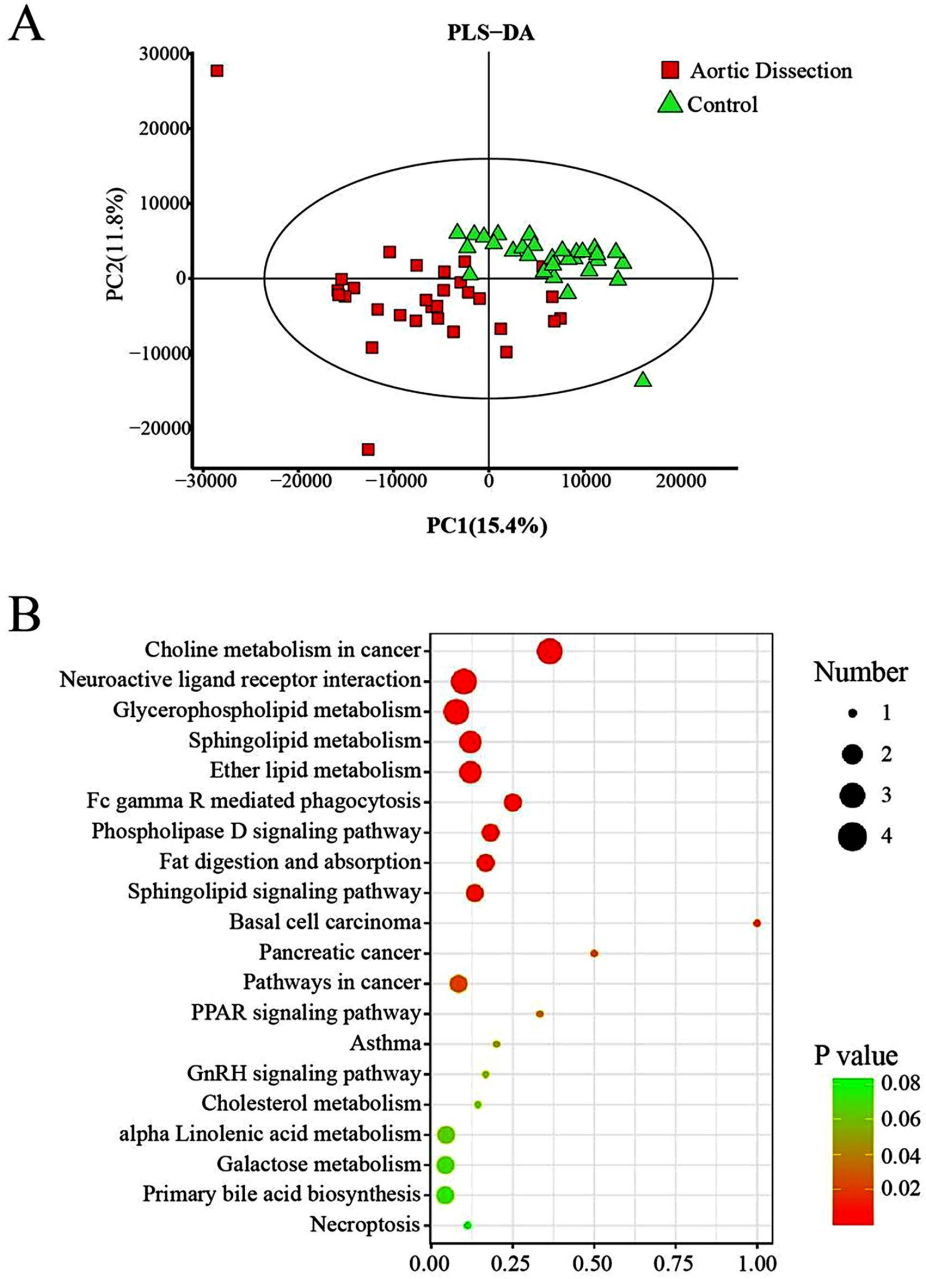
PLS-DA score plots and metabolic pathways

To reveal AD pathogenesis serum metabolites, the pairwise comparisons between the AD and HC groups were conducted. Based on the partial least squares (PLS-DA) method, the data showed significant separation between the two groups of samples (Fig. 2A) and the permutation test showed that the PLS-DA score were dependable without overfitting with R^2^ = 0 to 0.44 and Q2 = 0 to 0.455 (Supplementary materials, Fig. 2.)

**Fig. 3:**
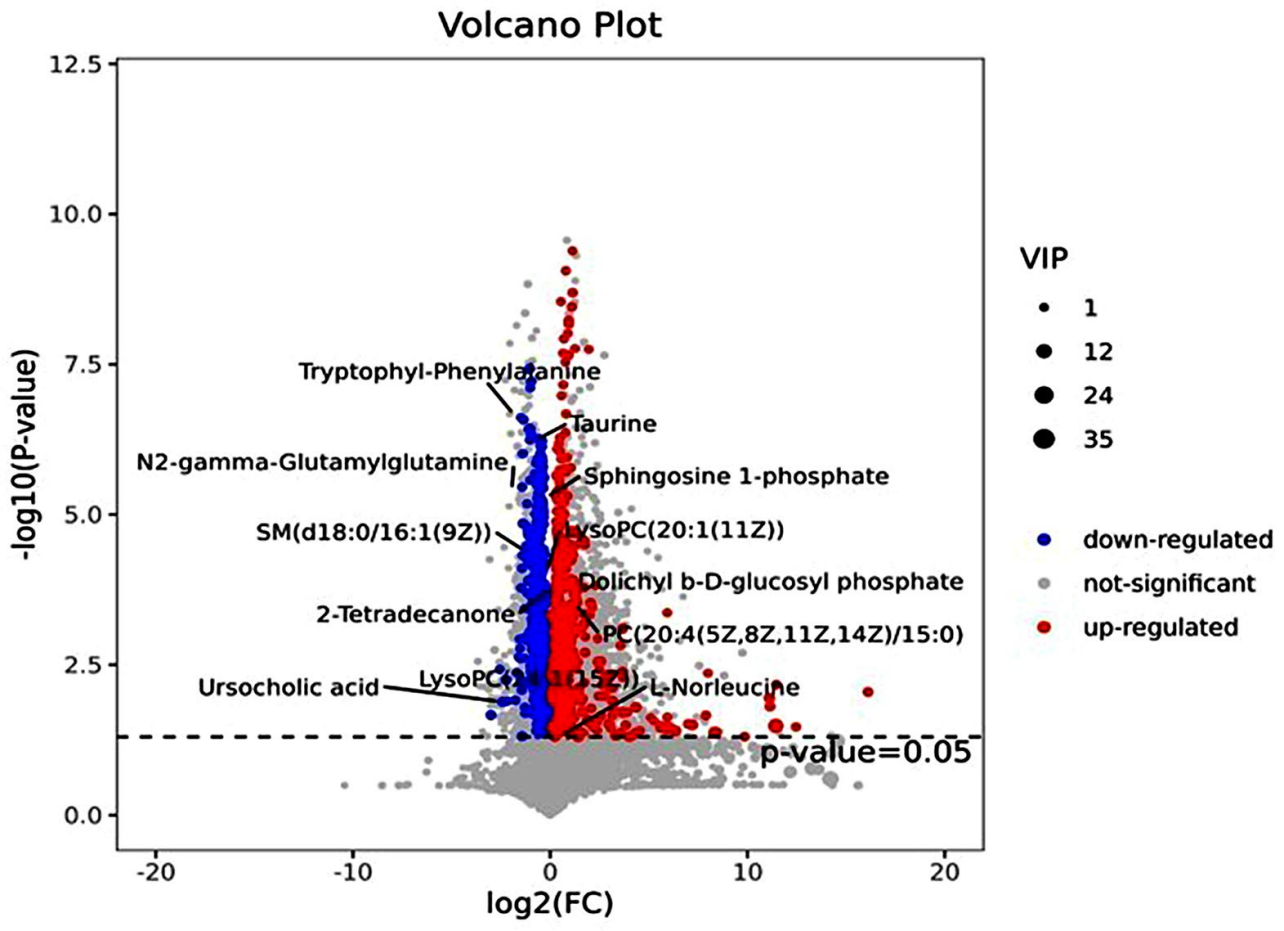
Thirteen specific metabolic biomarkers for distinguishing AD from HC

The screening criteria were that the variable’s importance in projection (VIP) > 1 and a *t*-test where *p* < 0.05. Once the metabolite fulfilled this condition, it was considered a potential biomarker. The AD and HC samples were compared to identify and characterize specific metabolites and underlying metabolic pathways. A total of 1221 metabolites, including 426 downregulated and 795 upregulated metabolites, were screened by LC-MS analysis and focused on 71 differential metabolites between two groups, Visualizing the p-value, VIP, and fold change values was helpful for screening differential metabolites, as shown in Fig. 3.

**Fig. 4:**
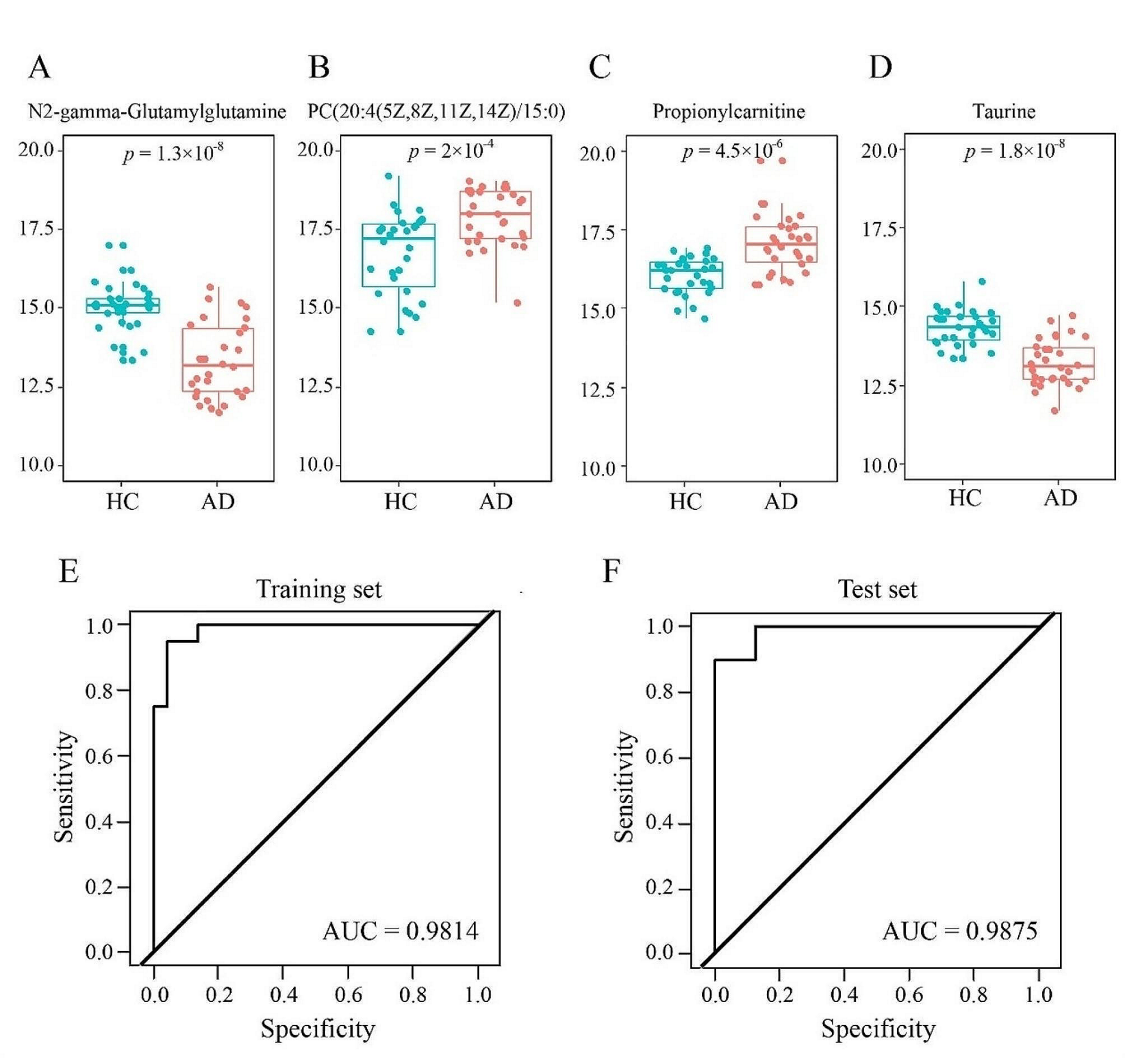
Diagnostic outcomes and prediction accuracies: the diagnostic outcomes are shown in the receiver-operating characteristic (ROC) curves for comparison between ADs and HCs. The prediction accuracies by the biomarkers in the training and test sets were compared between two groups

13 specific metabolic biomarkers were identified for distinguishing AD from HC (Table 2). A regression algorithm [6–9], least absolute shrinkage and selection operator (LASSO), was used to further identify four predictive metabolic biomarkers, i.e., N2-gamma-Glutamylglutamine, PC (20:4(5Z,8Z,11Z,14Z)/15:0), propionyl carnitine and taurine (Fig. 4), which served as a molecular diagnostic signature of AD. The training and test sets were set according to the concentration of metabolites and these four predictive metabolic biomarkers accurately classified the AD and HC samples with an AUC of 0.9875. Based on the value of the four different metabolites, a formula was developed to calculate the risk of aortic dissection:

$$\eqalign{{\rm{Risk}}\,{\rm{score}}\,{\rm{ = }} & \left( {{\rm{N2 - gamma - glutamylglutamine}}\,{\rm{ \times }}\, - {\rm{0}}{\rm{.684}}} \right) \cr {\rm{ }} & {\rm{ + }}\,\left( {{\rm{PC}}\,\left( {{\rm{20:4}}\,\left( {{\rm{5Z,8Z,11Z,14Z}}} \right)\,{\rm{/}}\,{\rm{15:0}}} \right)\,{\rm{ \times }}\,{\rm{0}}{\rm{.427}}} \right) \cr & {\rm{ + }}\,\left( {{\rm{propionyl}}\,{\rm{carnitine}}\,{\rm{ \times }}\,{\rm{0}}{\rm{.523}}} \right) \cr & {\rm{ + }}\,\left( {{\rm{taurine}}\,{\rm{ \times }}\, - {\rm{1}}{\rm{.242}}} \right) \cr}$$

As the circulatory levels of taurine and N2-gamma-glutamylglutamine are decreased, whereas, those of PC and propionylcarnitine are elevated in AD patients compared to HC subjects, Therefore, we use the Ratiometric to demonstrate it’s diagnostic potential.

R1 = PC(20:4(5Z,8Z,11Z,14Z)/15:0)/taurine = 1.8701/0.4862 = 3.85.

R2 = PC(20:4(5Z,8Z,11Z,14Z)/15:0)/N2-gamma-glutamylglutamine = 1.8701/0.3725 = 5.02.

R3 = propionylcarnitine/taurine = 2.3394/0.4862 = 4.81.

R4 = propionylcarnitine/N2-gamma-glutamylglutamine = 2.3394/0.3725 = 6.28.

**Table 2:** Statistical analysis of diagnostic biomarkers: discovery phase

Metabolites	log2(FC)	Retention time (min)	VIP	P-value	adj.P-value	FC
Dolichyl b-D-glucosyl phosphate	1.2323	4.9621	2.5299	0.000213	0.00424	2.3505
LysoPC(20:1(11Z))	-0.5924	12.3464	2.1241	8.21E-05	0.0024	0.66333
LysoPC(24:1(15Z))	0.2348	12.5863	1.2708	0.00223	0.01696	1.1768
PC(20:4(5Z,8Z,11Z,14Z)/15:0)	0.9031	14.104	2.4255	0.000332	0.005473	1.8701
SM(d18:0/16:1(9Z))	-0.9505	13.0766	19.0271	3.86E-05	0.001593	0.5174
Sphingosine 1-phosphate	-0.5039	10.3455	1.0297	4.92E-06	0.000451	0.7052
Taurine	-1.0405	0.68757	1.1709	5.74E-07	0.000128	0.4862
Ursocholic acid	-2.2665	9.5403	1.0286	0.01302	0.05564	0.2078
N2-gamma-Glutamylglutamine	-1.4247	0.6876	1.5007	3.49E-06	0.000364	0.3725
L-Norleucine	0.23482	1.3388	3.6752	0.04904	0.137118	1.1768
Propionylcarnitine	1.2261	1.3976	2.3843	0.000976	0.010334	2.3394
2-Tetradecanone	0.3418	8.8515	1.4916	0.00018	0.00387	1.2671
Tryptophyl-Phenylalanine	-1.4872	5.4163	1.0591	2.42E-07	7.86E-05	0.3567

**Fig. 5:**
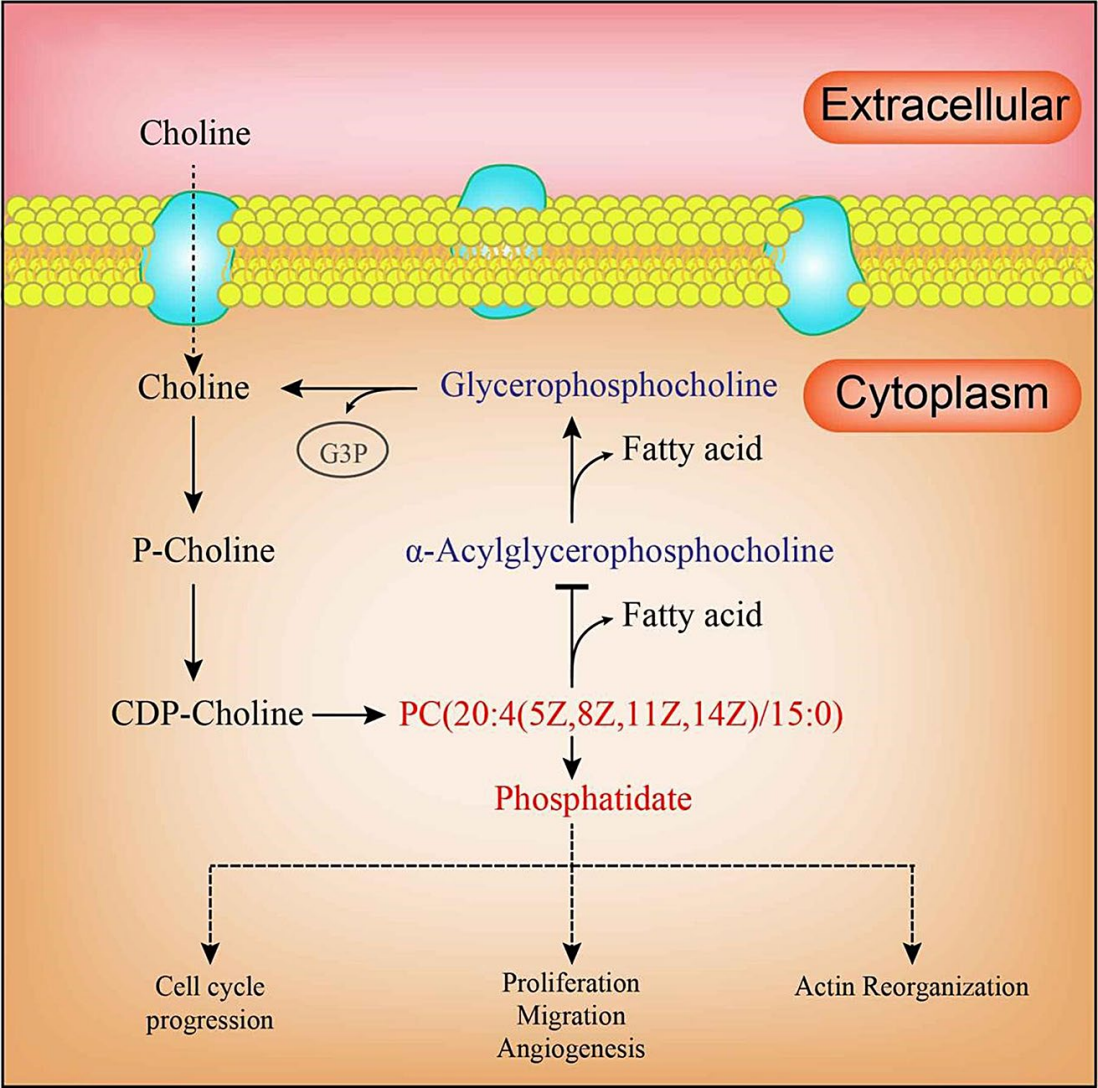
Proposed mechanisms of metabolite phosphitylated on pathogenesis of AD

To reveal the metabolic processes of metabolites and analyze the pathways in which these metabolites were involved, 71 differential metabolites were introduced into three Kyoto encyclopedia of genes and genomes (KEGG) databases HMDB, Lipimaps, and Metlin. The results suggested that the differential metabolites after screening were enriched in these three metabolic pathways choline metabolism in cancer, neuroactive ligand-receptor interaction and glycerophospholipid metabolism (Fig. 2B). The study found that of the metabolic biomarkers, PC (20:4(5Z,8Z,11Z,14Z)/15:0) played a key role in the glycerophospholipid metabolism pathway (Fig. 5) and PC (20:4(5Z,8Z,11Z,14Z)/15:0) activated the glycerophospholipid metabolism pathway downstream of phosphitylated, including cell-cycle progression, proliferation migration angiogenesis, and actin reorganization.

## Discussion

This study found that a metabolomic strategy could be used to identify potential metabolic markers of AD and to explore the metabolic pathways related to its occurrence. We carried out a comprehensive metabolomic evaluation of 30 AD patients. Metabolic phenotypes revealed significant pattern differences between patients with AD and HC, suggesting that AD may involve some metabolic disturbance, such as phospholipid metabolism disorder.

Patients with AD have a higher heart rate and elevated D-dimer levels [10]. . Except for heart rate at admission and D-dimer levels, the clinical baseline data were similar between the two patient groups .

Analysis of metabolic pathways enriched by 71 different metabolites showed that the different metabolites were involved in five pathways, including choline metabolism in cancer, neuroactive ligand-receptor interaction, glycerophospholipid metabolism, sphingolipid metabolism, and ether lipid metabolism. KEGG analysis showed oxidative stress and lipid transport and metabolism were significantly implicated.

Compared to HCs, patients with AD had down-regulated N2-gamma-glutamylglutamine(γ-glu) and taurine, both of which play a key role in amino acid and fat metabolism. especially taurine. There is evidence that it affects mitochondrial bioenergetics, counteracts lipid peroxidation and even increases cellular antioxidant defense in response to inflammation [11].

The patients with AD also had up-regulated propionyl carnitine and PC(20:4(5Z,8Z,11Z,14Z)/15:0). Propionyl carnitine is a free radical that can produce positive effects on endothelial function and protect endothelia from oxidative stress [12]. The increase in propionyl carnitine may be related to the repair of endometria after aortic dissection tear.

The PC(20:4(5Z,8Z,11Z,14Z)/15:0) is the precursor of phospholipids, which can decholine to produce phospholipids through a series of metabolic reactions in the body. The phospholipid metabolism disorder may be closely related to the occurrence of AD.

The diagnosis of acute AD relies on imaging such as vascular enhanced CT [13]. For community hospitals that lack relevant equipment, this is not conducive for the early diagnosis of acute aortic dissection, so finding a high-sensitivity metabolite of AD would be important for rescuing these patients.

This study identified and validated the signature consisting of four differential metabolites known to be related to AD pathogenesis that can accurately distinguish AD patients from healthy controls. Despite limitations such as the related small sample size, evidence showed that metabolites may be adopted as markers for the diagnosis of AD.

Aortic dissection results from a tear in the intimal layer of the aorta, leading to the separation of the layers of the aortic wall [14]. . While Extracellular matrix degradation and inflammation may contribute to the occurrence of the disease, the precise trigger of aortic dissections is still unknown [2]. Within the metabolic pathways enriched with differential metabolites, this study constructed a simple metabolic pathway model, wherein choline promoted the increase of phospholipids through transport proteins and second messengers. Phospholipids activated downstream pathways including cell-cycle progression, proliferation migration angiogenesis and actin reorganization, However, PC(20:4(5Z,8Z,11Z,14Z)/15:0) controls and regulates other pathways by negative feedback.

Previous studies considered that AD would activate cell-cycle progression [15, 16] and this is consistent with these results. The inflammatory response is deemed crucial for AD [17–19], inducing the expression of secretory molecules, including cytokines and extracellular matrix (ECM) molecules in smooth muscle cells (SMCs), thereby enhancing proliferative capacity [20]. Inflammatory cells, such as lymphocytes and macrophages, secrete multiple inflammatory cytokines to promote vascular adhesion molecule expression [21], eventually leading to proliferation migration angiogenesis. Inflammatory cells also contribute to the apoptosis of SMCs in the aortic artery and lead to medial degradation, exacerbating intimal tears [22].

Actin is an important protein, playing a critical role in many cellular functions and the interaction of actin with myosin forms the basis of muscle contraction [23]. In human aortic smooth muscle cells, smooth muscle α-actin (α-SMA) participated in filamentous actin formation and its expression can facilitate stress fiber formation and cell contraction in human aortic smooth muscle cells [24]. During the development of AD, smooth muscle cells may switch occurrence of phenotype [25],lead to actin reorganization and cause smooth muscle cell apoptosis [25], eventually causing the occurrence and progression of aortic dissection.

This study had some limitations. It failed to functionally verify the differential metabolites further. The sample size was small and could be further expanded in the future.

## Conclusion

In conclusion, we employed a metabolomics strategy to characterize metabolite signatures and their potential mechanisms associated with AD. Our results demonstrate that N2-gamma-Glutamylglutamine(γ-glu), taurine, propionyl carnitine, and PC (20:4(5Z,8Z,11Z,14Z)/15:0) were identified as critical metabolites. They may become markers for diagnosing aortic dissection. Moreover, phospholipids play a key role in the phosphorylated metabolite, leading to cell-cycle progression, proliferation, migration, angiogenesis, and actin reorganization. This evaluation enhances the understanding of AD pathogenesis.

## Data Availability

The data that support the findings of this study are available from the corresponding author upon reasonable request.

## Abbreviations

AD: Aortic Dissection
CT: Computed Tomography
HPLC: High Performance Liquid Chromatography
LC-MS: Liquid Chromatography-Mass Spectrometry
QC: Quality control
RT: Retention Time
PCA: Principal Component Analysis
LC-MS: Liquid Chromatography-Mass Spectrometry
(O) PLS-DA: Orthogonal Partial Least-Squares-Discriminant Analysis
HC: Healthy Controls
BMI: Body Mass Index
SMCs: Smooth Muscle Cells
ECM: Extracellular Matrix
α-SMA: Smooth Muscle α-actin
